# Measuring the effect of distance on the network topology of the Global Container Shipping Network

**DOI:** 10.1038/s41598-021-00387-3

**Published:** 2021-10-28

**Authors:** Dimitrios Tsiotas, César Ducruet

**Affiliations:** 1 Department of Regional and Economic Development, Agricultural University of Athens, Nea Poli, 33100 Amfissa, Greece; 2 French National Centre for Scientific Research (CNRS), UMR 7235 EconomiX, Nanterre, France

**Keywords:** Complex networks, Environmental economics, Sustainability

## Abstract

This paper examines how spatial distance affects network topology on empirical data concerning the Global Container Shipping Network (GCSN). The GCSN decomposes into 32 multiplex layers, defined at several spatial levels, by successively removing connections of smaller distances. This multilayer decomposition approach allows studying the topological properties of each layer as a function of distance. The analysis provides insights into the hierarchical structure and (importing and exporting) trade functionality of the GCSN, hub connectivity, several topological aspects, and the distinct role of China in the network’s structure. It also shows that bidirectional links decrease with distance, highlighting the importance of asymmetric functionality in carriers’ operations. It further configures six novel clusters of ports concerning their spatial coverage. Finally, it reveals three levels of geographical scale in the structure of GCSN (where the network topology significantly changes): the neighborhood (local connectivity); the scale of international connectivity (mesoscale or middle connectivity); and the intercontinental market (large scale connectivity). The overall approach provides a methodological framework for analyzing network topology as a function of distance, highlights the spatial dimension in complex and multilayer networks, and provides insights into the spatial structure of the GCSN, which is the most important market of the global maritime economy.

## Introduction

Spatial networks, and their underlying socioeconomic structures, are described by a symbiotic relation: on the one hand, networks facilitate trade and other socioeconomic interactions supporting regional and economic development^1,2^; on the other hand, the derived demand in the associated regional markets supports the development process of spatial and transportation networks, which are structures of considerable sunk costs affecting the future developmental dynamics of the spatial units participating in these networks^1^. Geography plays a crucial role in the development and evolution of spatial and transport networks. It is related to the friction of movements between places and interpreted in terms of transportation costs^1,3^. Due to spatial impedance, the connection probability between nodes usually decreases as distance increases^1–3^. In addition, new nodes entering a spatial network preferentially connect with nearby hubs, which already possess a high level of transport infrastructures (or amenities)^1,4,5^.

Therefore, the spatial property is determinative for the structure and functionality of networks and affects their organization, evolution and growth, topology, and traffic^2–4,6^. For instance, spatial networks are more likely to develop lattice-like than random, hub-and-spoke, or small-world topologies because spatial constraints usually impose planarity to these networks^3^. Among transportation networks, road, rail, and other “technical” networks are usually planar networks, whereas maritime and airline networks can exceed planarity and develop more complex hierarchical structures^3,7^. Also, in spatial networks, hubs are more likely to appear in central geographical places than in peripheral locations and to undertake most of the distant connectivity traffic. This property drives the correlation between node strength and degree linearly, at larger degree values^3,6^. In terms of functionality, spatial networks are usually quite different than those not embedded in space^3,6,8^. While, in social networks, nodes are actors directly operating and ruled by their behavioral and cognitive forces, spatial units—such as transport terminals or cities—function as the collective result of several actors^2^. Spatiality also favors the development of “regional” rather than “global” hubs, described by a smaller degree and much larger traffic than global hubs^3^. In terms of community detection, spatial constraints reinforce the geographical properties of community configuration, mainly driven by adjacency and neighborhood forces.

The spatial constraints are applicable at all levels of aggregation (neighborhood, local, global) in the network structure by introducing a distance cost in the development of connections^3^. However, their effect on network topology is not the same for all real-world networks as it depends on their functionality. For instance, empirical research^3,9–13^ has shown that road networks usually develop homogeneous and broadly invariant structures embedded in planar spaces. Due to planarity, severe constraints apply to (a) the node degree (that usually ranges around the average value ⟨k⟩ ≈ 2.5), (b) degree distribution (generally peaked around its average value), (c) average path length (expected to be large), and (d) clustering coefficient (that is larger than that of random counterpart networks). Depending on the representation (where nodes usually represent road intersections or spatial units) and geographical scale, road networks are usually connective graphs described by mesh-like and lattice-like topologies relevant to two-dimensional lattices. Accordingly, railway networks^3,14–17^ usually are embedded in planar spaces, with similar topology to that of road networks, but vary in terms of nodes (stations, stops, or spatial units) and linear arrangements (bus-like structures). On the other hand, maritime networks^18^ can display different properties than land transportation networks and overcome planarity due to their attribute to conduct transportation on the sea surface instead of line infrastructure channels. This structural property is reflected^3,7,18–23^ on (a) higher average degree than land transportation networks, (b) much larger average clustering coefficient than of counterpart lattices, and (c) degree distributions described by power-law patterns that often are typical of standard non-spatial networks. Depending on the representation (where nodes usually represent ports) and geographical scale, maritime networks are not by default connective graphs (where components are sub-networks representing local markets) and are described either by composite ring-like or hub-and-spoke topologies. Amongst the various types of maritime transport, cargo ship networks^7,8,21,24–26^ have received much attention in the literature due to their economic importance, as more than 80% of world trade volumes are carried by sea. In such networks, the weight configuration is very asymmetric (w_ij_ ≠ w_ji_), with almost 60% of all linked pairs existing only in one direction. This condition is mainly because container shipping is operated through asymmetric pendulum services with different legs back and forth within and between regions^24^. Finally, airline networks are non-planar spatial networks, privileged to conduct transportation in the 3d-space instead of line infrastructure channels. This property reflects on^3,7,27–33^ (a) the shape of the degree distribution, which fits power-law curves and is heavy-tailed with a cutoff, due to the physical constraints on the maximum number of connections that a single airport can handle; (b) the average path length, which is smaller than of counterpart lattices; (c) the significant correlations between topology and geography, implying that larger airports have connections with higher traffic and also more distant connections; (d) the high clustering, which is slightly decreasing at large degrees (*k*), illustrating the role of large airports that provide non-stop connections to different not interconnected regions; (e) and a clique-like configuration, because large weights concentrate on links between large airports. Depending on the representation (where nodes usually represent airports) and geographical scale, airline networks are also not by default connective graphs. They usually have hub-and-spoke topology with heterogeneous topological properties, where hubs undertake high connectivity, large traffic load, and long-distance connections. Airline networks emerge at two different geographical scales^3^, global and domestic, where the first one defines two different groups of travel distances (intercontinental and regional).

Besides the complexity describing its ontology, the relationship between space and network topology submits to different disciplinary foci^2,6^. For instance, economists and regional scientists tend to conceptualize space in econometric terms and to manage the spatial dimension either as a separate econometric variable or through a given cost or facilitation (such as trade cost, tariff, and time); geographers, engineers, and spatial planners consider space as geospatial information expressed by geographical coordinates and territorial attributes (such as port city population, socioeconomic features, etc.); while physicists and mathematicians analyze the topological differences captured before and after the networks embedding in metric spaces^2,6^. Despite this polyphony, it is possible to synthesize the literature on spatial networks and related constraints as follows^2,3,6,33^: (a) through the examination of the shape of degree distribution, where a bell-shaped configuration peaked around the average degree implies the effect of planarity in the configuration of network topology; (b) in reference to theoretical (null) graph models, according to which topological aspects and measures of real-world or other empirical networks are compared with the results of counterpart null models of lattice-like, random-like, ring-like, small-world, hub-and-spoke, and other known topologies; (c) sometimes through spectral pattern recognition based on the sparsity (spy plot) patterns of the adjacency and connectivity matrices of spatial networks, and (d) by examining correlations between the measure of degree and the betweenness centrality (i.e. network intermediacy) and strength (i.e. traffic volume), to detect whether hubs (i.e. nodes of high connectivity) also intermediate to the majority of paths and undertake the highest traffic load.

Although very informative and insightful, existing methods generally study the relationship between space and network topology within a holistic and structurally undisturbed context, thus conceptualizing networks as integrated and indivisible structures. This conceptualization is quite informative when networks represent systems of socioeconomic interaction^1–3^, where each one has a single balanced macroeconomic behavior. However, networks also include elements of diverse importance and functionality (such as hubs, spokes, strong and weak ties, etc.) that are often hierarchically organized^3^. Decomposition techniques in the research fields of community detection^33^ and vulnerability analysis^21^ are not yet applied to unravel the relationship between space and network topology.

In such respect, this paper decomposes a spatial network of outstanding economic importance, the Global Container Shipping Network (GCSN), by successively removing edges (links) of shorter distances and by assigning the remaining links into layers. It thus configures a multilayer network model consisting of a family of layers, where each one includes links longer than a certain distance. This approach allows configuring networks of distinct topologies for edge-groups of different spatial ranges; and thus attempts to provide insights into the effect of space on the topological features of a spatial network, at diverse levels of spatial scale. To this aim, the analysis builds on multilayer network modeling^21,34,35^, which allows describing a system as a collection of layers instead of a single network. Some indicative approaches on multilayer analysis of spatial networks include analysis of air-sea global networks^36^, the bi-layered Greek maritime network^37^, and the intermingling of six maritime cargo layers^25^. These and similar works^21,38–40^ generally build on a cross-layer conceptualization implemented through comparisons between multilayer spatial network layers. Inspired by such conceptualization, this paper generates a multilayer network from a source (original) network, where layers include the same nodes (multiplex network model) with the original network. However, each layer contains a subset of the original links exceeding a certain distance. This multiplex network model is a consistent collection of layers where the topological features can express a function of distance along the cross-layer direction (axis).

The analysis in this paper is applied to the Global Container Shipping Network (GCSN), first because it is a network of prominence economic importance, consisting of international trade flows of all over the globe^8,25^. Maritime transportation is the dominant mode of transport in international trade. Around 80% of global trade volume and over 70% of global trade value are transported by sea and handled by ports^21^. In the last half-century, the spatial characteristics of maritime networks changed tremendously^1^. In particular, the GCSN has a complex structure driven by the counterbalance between spatial distance, market forces, and technology, which is related to (a) the transportation cost between places^2,41^; (b) the availability of container transportation, which lowers the effects of distance due to massification^41^; (c) the increase of trade demand due to urbanization and global manufacturing shift^25^; (d) the changing composition of trade and the increasing containerization of all sorts of commodities of which bulks and even automobiles^42^; (e) the growth in the size of container ships achieving economies of scale^25,41^; and (f) the transformation and liberalization of the maritime market, which changed from a labor-intensive to a capital-intensive industry^18,26^.

Also, the global shipping network is an assemblage of different types of liner shipping services, each characterized by a varying degree of shipping distance^43^. Deep-sea services occur over longer edges and include round-the-world and pendulum services deployed on inter and intra-continental legs. Short-sea services occur over shorter edges and include feeder services between hub and spokes, coastal shipping, and the short-sea service itself usually bound to a closed sea or within a maritime range^44^. The interlining service is a specific transshipment activity type whereby two deep-sea services exchange containers at a given hub. The emergence of transshipment hubs in the mid-1990s led to the increasing use of feeder services on short distances, while the progress of regional integration favored the multiplication of short-sea shipping (see^45^). Concrete examples of transshipment hubs include ports with a high ratio of sea-sea transshipment, such as Singapore, Salalah (Oman), Gioia Tauro (Italy), and Kingston (Jamaica), often located within a maritime basin with the minimum deviation distance from the trunk line^46^. Intra-continental pendulum services deploy through multiple calls among ports in proximity, such as at the Northeastern seaboard of America, the Le Havre-Hamburg range in North Europe, and the Japanese megalopolis from Fukuoka to Tokyo. The increasing ship size (up to the present era of mega-ships) motivated longer voyages and fewer port calls. Lastly, the GCSN is specific as vessels need to avoid coastlines.

Such elements motive this paper to question whether (a) rapid technological improvements, (b) increased globalization, and (c) lowering costs contributed to modify (or even nullifying) the effect of distance on the topological structure of the GCSN. On the one hand, recent research demonstrated the persistence of gravitational properties affecting the GCSN^18^, as large (port) cities interconnect more but less over longer distances. Such a result is in line with international trade studies examining the “puzzling effect of distance” on bilateral exchanges^47^, one of the most important findings in economics. On the other hand, the introduction of containers is believed to lowered distance impacts since the 1980s^48^, an effect which had, however, been challenged by China’s trade growth^49^. While the search for distance effects in networks now has a long tradition in physical^50,51^ and social^52,53^ sciences, the existing literature usually considers the network as one single, aggregated entity. This paper goes one step further by decomposing the network into layers of varying link distance. Such an angle of attack has the merit to highlight the ability of ocean carriers to overcome distance and to better understand the centrality and functionality of hubs, from the local to the global.

The remainder of the paper organizes as follows: the methods section describes the modeling and the methods used for the analysis of the GCSN; the next section shows the multilayer GCSN network and empirical analysis results; and finally, the last section gives the conclusions.

## Methods

### Conceptual framework, modeling, and data

The Global Container Shipping Network (GCSN) is modeled to a multilayer graph ${\mathcal{M}}$ (${\mathcal{G}}$, ${\mathcal{C}}$ = Ø)^34,35^. In GCSN ${\mathcal{G}}$ =  {*G*_o_, *G*_1_, *G*_2_, …, *G*_31_} is a family of 32 layers generated from the original layer *G*_o_, and ${\mathcal{C}}$ =  Ø is the null set of interlayer connections (implying that all GCSN connections are within-layer links and no link between layers exist). The original layer *G*_o_(*V*,*E*) was constructed on the annual direct routes between the GCSN ports and particularly on 282,785 movements of fully cellular container vessels in 2016. Ports are nodes (belonging to the set *V* | $\left| V \right| = n$) and links (belonging to the set *E* | $\left| E \right| = m$) inter-port voyages^18,43^. The GCSN has a dual weighted representation: the first one is distance-weighted ($w_{ij}^{(a)} = d_{ij}$), where links are weighted by their orthodromic distance, namely by the geographical length of a straight line considering the rotundity of the Earth, and measured in nautical miles (1 nm = 1.852 km); the second one is freight-mass-weighted ($w_{ij}^{(b)} = w_{ij}$), where edge weights represent annual carried freight mass expressing the cumulated vessel capacity measured in deadweight tons (DWT). The total DWT of a link corresponds to the product between voyages and vessel capacities^25^. The data used in this study was obtained from Lloyd’s List Intelligence, a world leader in maritime insurance and information, which tracks the daily movements of more than 80% of the world fleet and 98% of the world container fleet^18,25^.

The original layer (*G*_o_) of the multilayer GCSN (${\mathcal{G}}$) is a directed graph composed of *n* = 1109 nodes (ports of worldwide container connectivity) and *m* = 12,069 links (direct container connections between ports). The other 31 layers are also directed and are induced from *G*_o_ $\in$ ${\mathcal{G}}$ by sequentially removing edges of shortest distances. In particular, the first layer (*G*_1_) is composed of the same number of nodes as the original layer (*n*_1_ = *n*_o_), but includes only those edges having a greater distance than 100 nm, namely *G*_1_(*V*_1_, *E*_1_) = *G*(*V*, *E* ≥ 100 nm). Similarly, the other layers are generated by applying the respective restrictions *E* ≥ 200; 300; 400; 500; 600; 700; 800; 900; 1000; 1250; 1500; 1750; 2000; 2500; 3000; 3500; 4000; 4500; 5000; 5500; 6000; 6500; 7000; 7500; 8000; 8500; 9000; 9500; 10,000; and 10,500 nm.

**Figure 1:**
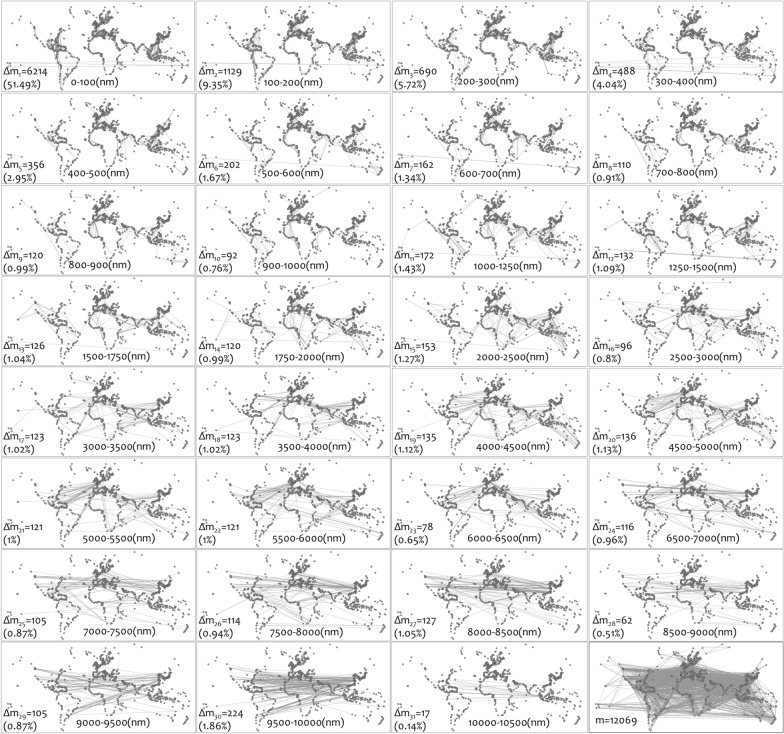
Edges (Δ*m*_*i*_) added in each layer of the multilayer GCSN (*n* = 1109 nodes, *m* = 12,069 edges). The edges *m*_*i*_ included in layer *G*_*i*_ are those with distance shorter than ( ≥) *i* nm, where: *i* = 100; 200; 300; 400; 500; 600; 700; 800; 900; 1000; 1250; 1500; 1750; 2000; 2500; 3000; 3500; 4000; 4500; 5000; 5500; 6000; 6500; 7000; 7500; 8000; 8500; 9000; 9500; 10,000; 10,500 (data from the year 2016); and are computed by the formula *m*_*i*_ = *m*_*i–*1_ + Δ*m*_*i*_ (*m*_*o*_ = 0).

Let us denote this set of spatial distances as $${\mathcal{S}}$$. This method of layer construction builds on an unevenly stratified distance sampling applying: (a) a step of 100 nm to the interval [100–1000]; (b) a step of 250 nm to the interval [1000–2000]; and (c) a step of 500 nm step to the interval [2000–10,500]. The concept behind this unevenly stratified sampling is to empirically counterbalance data resolution, computational complexity, sample adequacy for statistical inference analysis, and empirical intuition in the available family of layers. In particular, while it is desirable to reduce the resolution of the layers' dataset and to ease computations, the available number of layers firstly should be also enough (> 30) to apply statistical inference analysis based on the normality assumption (to construct normal distribution confidence intervals)^54^. Secondly, the level of resolution should be satisfactory enough at the shorter (neighborhood) distances, where the major volume of cargo activity^18,21,25^ is by definition applicable. The property *V*_0_ = *V*_1_ = *V*_2_ = … = *V*_31_ = *V* makes $${\mathcal{G}}$$ a multiplex network, where all layers have the same number of nodes and the edge-set (*E*_*i*_) in each layer is produced by applying a distance filter (*E* ≥ *d*(*i*) nm | *d*(*i*)$$\in$$ $${\mathcal{S}}$$, *i* = 0,1,2,…,31). Let Δ*E*_*i*_ denote the edge difference (Δ*E*_*i*_ = *E*_*i*_ − *E*_*i*–1_ | *i* = 1,2,…,31) between layers *G*_*i*–1_ and *G*_*i*_ (see Fig. 1). Thereby, we can define each layer *G*_*i*_ $$\in$$$${\mathcal{G}}$$ as a function of the original one *G*_*i*_ = *f*(*G*_o_), as follows:

1 $$G_{i} \left( {V_{i} ,E_{i} } \right) = G_{o} \left( {V,E(i)} \right) = G_{o} \left( {V,E - \sum\limits_{j = 1}^{i} {\Delta E_{j} } } \right)$$

The definition context of the GCSN’s layers illustrates that this study does not conceptualize spatial distance as a primary variable to measure its effect on network topology. Instead, it conceives spatial distance indirectly, through the criterion applied to generate the network layers of ${\mathcal{G}}$. In particular, we assume in this study an (unknown) underlying relationship between the topological features with the spatial constraints of each layer. This assumption stands because (a) each layer is a separate network of characteristic topological properties, and (b) spatial constraints are determinants to the network configuration. Therefore, this paper conceives spatial distance through its transformation into the topological properties of layer *G*_*i*_ produced under specific spatial constraints *d*(*i*)$\in$ ${\mathcal{S}}$. So, if we denote as ${\mathcal{X}}$ the topological space where the original layer *G*_o_ is embedded, we can thus define the topological space ${\mathcal{X}}$_*i*_ ≡ ${\mathcal{X}}$(*i*) ≡ ${\mathcal{X}}$(*d*(*i*)) of layer *G*_*i*_ as a function of ${\mathcal{X}}$ and distance *d*(*i*)$\in$ ${\mathcal{S}}$. Partially, this definition implies that ${\mathcal{X}}$(*i*) is a function of distance. Within this context, we loosely define the topological space ${\mathcal{X}}$(*i*) as what in literature is called network topology. Many academics prefer to use a single characterization to describe this property, such as random-like, lattice-like, small-world, and hub-and-spoke topology^2–4^. In this paper, we define network topology ${\mathcal{X}}$(*i*) in vector terms. In particular, ${\mathcal{X}}$(*i*) is composed of a series (*x*_1_, *x*_2_, …, *x*_*p*_) of fundamental topological measures and attributes, as follows:

2 $${\mathcal{X}}\left( i \right) = {\mathcal{X}}_{{\text{i}}} \left( {x_{{1}} ,x_{{2}} , \ldots ,x_{p} } \right) = {\mathcal{X}}(x_{{1}} (i),\;x_{{2}} (i), \ldots ,x_{p} (i)),$$

where each component *x*_1_, *x*_2_, …, *x*_*p*_ describes the topological features of one layer *G*_*i*_.

This approach allows studying network topology in a multivariable context, as the collection of various measurable attributes, and not with a single topological characterization. Besides, the GCSN has a known hub-and-spoke network topology with scale-free characteristics^18,20,24,25^ and thus a mono-variable consideration of its network topology (based on the power-law distribution exponent or a categorical network topology variable) would not be much insightful. On the other hand, this paper's multivariable context incorporates in more detail topological information. This is done by decomposing topological space ${\mathcal{X}}$(*i*) into a set of measurable topological attributes ${\mathcal{X}}$(*i*) =  ${\mathcal{X}}$(*x*_1_(*i*), *x*_2_(*i*), …, *x*_*p*_(*i*)). Thereby, this paper conceives spatial distance not as a single geographical variable but through the effect of the spatial constraints (*E* ≥ *d*(*i*)) on the topological attributes ${\mathcal{X}}$(*x*_1_(*i*), *x*_2_(*i*), …, *x*_*p*_(*i*)) of the GCSN layer *G*_*i*_, for the GCSN layers' construction *G*_*i*_(*V*, *E* ≥ d(i)). The overall approach goes beyond the typical understanding of network topology and contributes to its multivariable conceptualization.

### Network and empirical analysis

This paper studies a set of topological properties ${\mathcal{X}}$(*i*) =  ${\mathcal{X}}$(*x*_1_(*i*), *x*_2_(*i*), …, *x*_*p*_(*i*) | *i* = 0,1,2,…,32) on the available family = ${\mathcal{G}}${*G*_o_, *G*_1_, *G*_2_, …, *G*_31_} of the 32 GCSN layers. The rationale behind this approach bases on the research hypothesis that important connections are determinant to the configuration of network topology and therefore network topology should considerably change when important connections omit from the network. Taking into account that each layer is generated under a spatial constraint *E* ≥ *d*(*i*), with *d*(*i*)$\in$ ${\mathcal{S}}$, the scores’ collection for a topological measure *x*_*j*_ $\in$ ${\mathcal{X}}$across the GCSN layers {*x*_*j*_(0), *x*_*j*_(1), *x*_*j*_(2), …, *x*_*j*_(32)} configures a series expressing the behavior of *x*_*j*_ for different distances {*d*(0), *d*(1), *d*(2), …, *d*(32)}. This allows first considering each topological measure *x*_*j*_ $\in$ ${\mathcal{X}}$ as a function of distance *x*_*j*_ = *x*_*j*_(*d*(*i*)). Secondly, it allows interpreting the behavior across the different distance constraints as the effect of distance on the topological measure *x*_*j*_ $\in$ ${\mathcal{X}}$. Further, the collections {*x*_*j*_(0), *x*_*j*_(1), *x*_*j*_(2), …, *x*_*j*_(32)} for all topological measures *j* = 1,2,…,*p* can provide an approximation of the effect of space on network topology. However, although successful in describing the topological properties of the GCSN layers as a function of distance, this approach does not suggest a typical case of “distance attenuation law”^1,3^. This differentiation is due to the way distance is conceived, namely through the transformation of spatial constraints to the topological properties of GCSN layer (and not as a direct variable). Within this framework, we include in the analysis a set of network measures described in Table 1, retrieved from the relevant literature^4,55–57^.

**Table 1:** Network measures ^(^*^)^ included in the analysis of the GCSN.

Measure	Symbol	Description	Math formula
*Graph density*	*ρ*	The fraction of the existing graph connections (*m*) to the number of the possible connections $$\left( {\begin{array}{*{20}c} n \\ 2 \\ \end{array} } \right)$$, where: *n* is the number of nodes. It expresses the probability to meet in the GMN a connected pair of nodes	$$\rho = {m \mathord{\left/ {\vphantom {m {\left( {\begin{array}{*{20}c} n \\ 2 \\ \end{array} } \right)}}} \right. \kern-\nulldelimiterspace} {\left( {\begin{array}{*{20}c} n \\ 2 \\ \end{array} } \right)}} = \frac{2m}{{n \cdot (n - 1)}}$$
*Node Degree*	*k*	The number of edges *k*(*i*) adjacent to a given graph *G*(*V*,*E*) node i, where: *V* is the node-set and *E* is the edge-set. Node-degree expresses the node’s communication potential	$$\begin{gathered} k_{i} = k(i) = \sum\limits_{j \in V} {\delta_{ij} } ,{\text{ where}} \hfill \\ \, \delta_{ij} = \left\{ {\begin{array}{*{20}c} {1,{\text{ if }}e_{ij} \in E} \\ {0,{\text{ otherwise}}} \\ \end{array} } \right. \hfill \\ \end{gathered}$$
*Node strength*	*s*	For a network edge $e_{ij} \in E$, (where *E* is the edge-set) node strength *s*(*i*) is the sum of edge weights *w*_*ij*_ adjacent to a given node *i*	$s_{i} = s(i) = \sum\limits_{j \in V} {\delta_{ij} \cdot w_{ij} }$
*Average Path Length*	$\left\langle l \right\rangle$	The average length of the network shortest-paths *d*(*i*,*j*); *n* is the number of nodes in the network	$\left\langle l \right\rangle = \frac{{\sum\limits_{i \in V} {d(i,j)} }}{n \cdot (n - 1)}$
*Network diameter*	*d*(*G*)	The maximum length of the network shortest-paths *d*(*i*,*j*)	$d(G) = \max \left\{ {d(i,j)} \right\}_{i,j \in V}$
*Clustering Coefficient* (*local*)	*C*(*i*)	The probability of meeting linked neighbors around node *i*. It is equivalent to the number of the node's connected neighbors *E*(*i*) (i.e., the number of triangles in the neighborhood), divided by the number of the total triplets shaped by this node, which equals to *k*_*i*_(*k*_*i*_–1), where: *k*_*i*_ is the degree of node *i*	$C(i) = \frac{E(i)}{{k_{i} \cdot \left( {k_{i} - 1} \right)}}$
*Modularity*	*Q*	An objective function expressing the potential of a network to be subdivided into communities, where: *g*_*i*_ is the community of node *i* $\in$ *V* (*V* is the node-set), [*A*_*ij*_ – *P*_*ij*_] is the difference of the actual (*A*_*ij*_) minus the expected (*P*_*ij*_) number of edges falling between a particular pair of vertices *i*,*j* $\in$ *V*, and *δ*(*g*_*i*_,*g*_*j*_) is an indicator (the Kronecker’s) function returning 1 when *g*_*i*_ = *g*_*j*_	$Q = \frac{{\sum\limits_{i,j} {[A_{ij} - P_{ij} ] \cdot \delta (g_{i} ,g_{j} )} }}{2m}$

*Sources: ^4,55–57^.

Within this context, the methodological approach in this paper applies toward a double direction. The first builds on the conceptualization of the current methods examining the relationship between space and network topology within a holistic and structurally undisturbed context. This direction performs an analysis on the original layer (*G*_o_) of the GCSN to detect topological properties related to the effect of space. The second direction builds on the decomposition rationale developed in this paper, examining the collections of the available topological measures across the 32 layers of the multilayer GCSN as a function of distance (as previously were described in detail).

In technical terms, the methods used in the analysis are first the computation of the degree distribution *p*_*i*_(*k*) of each layer *G*_*i*_, expressed by the frequency distribution (*k*_*j*_, *n*(*k*_*j*_)) of the unique node degree values *k*_*j*_ in the network, as follows^3,4,58^:

3 $$p(k) = \left( {k_{i} ,n\left( {k_{i} } \right)} \right)$$

where *n*(*k*_*i*_) is the node frequency (number of nodes) of degree *k*. When node frequencies divide by the total number of nodes (*n*(*k*_*i*_)/*n*), the degree distribution interprets the probability to meet a node of degree *k*_*i*_. Further, both distance and deadweight tonnage (cumulated vessel capacity) edge distributions are computed by constructing histograms^54,59^, expressing the edge frequency within classes of ranges of edge weights.

Next, parametric curve fittings apply to degree distributions and the series of topological measures as a function of distance. This process estimates the parameters of a curve *y* = *f*(*x*) that optimally fit to the observed data *y*_*i*_ by minimizing the square differences ${e=y}_{i}-\widehat{{y}_{i}}$, according to the relation^54,59^:

4 $$\min \left\{ {e = \mathop \sum \limits_{i = 1}^{n} \left[ {y_{i} - \hat{y}_{i} } \right]^{2} } \right\} = \min \left\{ {\mathop \sum \limits_{i = 1}^{n} \left[ {y_{i} - f(k_{i} )} \right]^{2} } \right\}$$

The optimization method used for the estimations is the Least-Squares Linear Regression (LSLR), which bases on the assumption that differences *e* follow the normal distribution *N*(0,${\sigma }_{e}^{2}$).

Next, on the series of (*p* in number) topological measures {*x*_*j*_(0), *x*_*j*_(1), *x*_*j*_(2), …, *x*_*j*_(32) | *j* = 1,2,…,*p*} we compute the first-order differences, according to the formula^60,61^:

5 $$\Delta^{(1)} x_{j} (i) = x_{j} (i) - x_{j} (i - 1),\;i = {1}, \ldots ,{32,}$$

where *x*_*j*_(*i*) is the value of the attribute-series *x*_*j*_ at a place (distance) *i*, and *x*_*j*_(*i*–1) at the previous position *i*-1. This approach expresses the discrete analogy of the first derivative function and allows capturing the changes in a network attribute without removing the variable’s scale. After the computation of the first-order differences, we statistically test whether their values are significantly higher than the average (mean value) of the first-order series. To do so, we construct 95% confidence intervals (CIs) of the mean, according to the formula:

6 $$\mu_{lb,ub} = \hat{\mu } \pm z_{a/2} \cdot se,$$

where $\hat{\mu }$ is the sample estimator of the mean value, *z*_*α*/2_ is the z-score computed for 1–*a*% confidence level, and $se = {s \mathord{\left/ {\vphantom {s {\sqrt n }}} \right. \kern-\nulldelimiterspace} {\sqrt n }}$ is the sample’s standard error^54^. Cases exceeding the (zone) interval [*μ*_*lb*_, *μ*_*ub*_] are considered statistically different from the mean value and thus imply that the attribute-series exhibit significant changes.

To detect whether (a) connectivity in hubs is an effect of distance and (b) at what level the hubs undertake the distant connections of the GCSN, we examine the attribute-series of the measures of (a) degree (*k*), (b) in-degree (*k* +), and (c) out-degree (*k*–). The analysis applies to the Top 10 highly connected ports (hubs) of the original layer, along with some other chosen ports that showed considerable constancy to distance, as shown in Table 2.

**Table 2:** Top 10 hubs (ports with the highest connectivity) and selected ports included in the analysis of the GCSN.

Degree			In-degree			Out-degree		
Rank	Port	Measure	Rank	Port	Measure	Rank	Port	Measure
1	Singapore	345	1	Singapore	170	1	Singapore	175
2	Busan	300	2	Busan	148	2	Busan	152
3	Shanghai	247	3	Shanghai	135	3	Hong Kong	120
4	Hong Kong	246	4	Rotterdam	128	4	Rotterdam	116
5	Rotterdam	244	5	Hong Kong	126	5	Port Klang	115
6	Port Klang	233	6	Port Klang	118	6	Shanghai	112
7	Algeciras	206	7	Algeciras	106	7	Algeciras	100
8	Tanjung Pelepas	180	7	Qianwan	106	8	Tanjung Pelepas	93
9	Qianwan	174	8	Antwerp	98	9	Jebel Ali	82
10	Antwerp	167	9	Beilun	87	10	Beilun	78
11	Beilun	165	9	Kaohsiung	87	13	Kaohsiung	74
12	Kaohsiung	161	9	Tanjung Pelepas	87	17	Antwerp	69
12	Jebel Ali	161	12	Jebel Ali	79	17	Yangshan	69
16	Yangshan	147	13	Yangshan	78	18	Qianwan	68
28	New York	115	19	New York	66	28	New York	49

Finally, we apply a factor analysis on edge distribution (DWT) variables configured by percentile of link distance to detect commonalities that may provide insights into the hierarchy of GCSN due to the effect of space. This method describes the variability among possibly correlated variables by the variability reflected on a potentially lower number of unobserved variables, called factors (underlying or latent variables or components). Factor analysis models the observed variables as linear combinations of the potential factors, including stochastic terms, and generally contributes to the dimension reduction of the observed data. The method applies an orthogonal transformation to the original variables, like fitting a *p*-dimensional ellipsoid to the data, where each axis of the ellipsoid corresponds to a factor. Dimension reduces when some axes of the ellipsoid are relatively small, implying that the variance along that axes is also small so that these axes can omit from the dataset^59^.

## Results

### Degree and edge distributions

**Figure 2:**
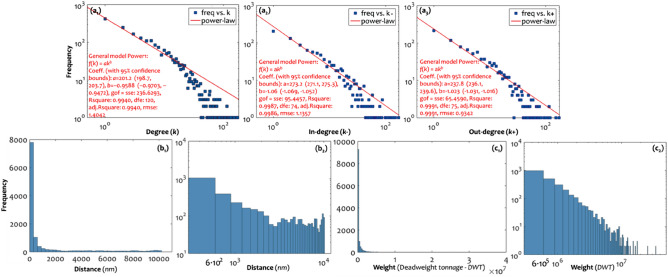
(**a**_**1**_) Degree (*k*, *n*(*k*)), (**a**_**2**_) In-degree (*k*–, *n*(*k*–)), and (**a**_**3**_) In-degree (*k* + , *n*(*k* +)) distribution, showing the edge (distance-weighted, measured in nautical miles—nm) distribution at (**b**_**1**_) metric and (**b**_**2**_) log scale, and histograms showing the edge (mass-weighted, measured in deadweight tonnage—DWT) distribution at (**c**_**1**_) metric and (**c**_**2**_) log scale, of the original layer (*G*_o_) of the GCSN.

The GCSN was extensively studied in the literature as a complex network^7,8,21,24–26^, due to its prominence economic (and particularly trade) importance. In all different available aspects of this cargo shipping network in the literature, scale-free characteristics are evident in the power-law (PL) fittings of its degree distribution, despite that they do not suggest a typical case^4^ of a scale-free network. This analysis, applied to the degree and edge distributions of the GCSN according to the first methodological dimension structurally undisturbed context, supports existing literature findings. In particular, in Fig. 2, we can observe that all undirected (*k*), in-degree (*k*–), and out-degree (*k*+) distributions of the original GCSN layer (*G*_o_) strongly fit PL patterns, with high scores of coefficient of determination *R*^2^(*k*) = 0.994, *R*^2^(*k*) = 0.9987, and *R*^2^(*k*) = 0.9991, respectively. In addition, although they do not fall within the typical interval of scale-freeness (2 < *γ* < 3), the slopes of in-degree and out-degree distributions are significantly (at 95% confidence level) greater than one (> 1). This result illustrates a tendency of the GCSN to configure structures of hierarchy (see^3,4,58^) in its incoming (related to import trade activity) and outgoing (related to export trade activity) connectivity. On the other hand, the PL exponent of the undirected degree distribution is significantly (95%) lower than one (< 1), describing a smoother slope of PL decay in comparison with the directed cases. These results indicate that hubs in the undirected representation of the GCSN are more in number (frequency) compared with the directed representation, a fact that reveals the asymmetric functionality in carriers’ operations of the GCSN.

A similar PL-like picture is also evident at the edge distributions in Fig. 2. In particular, both the distance and deadweight tonnage (DWT) edge distributions of the GCSN configure distinct PL patterns. On the one hand, the edge distribution of distance is long-tailed, with most of the connections being shorter than 1000 nm. In the log–log scale, we can observe a change in the edge distribution slope for distances greater than 3000 nm, where, in the metric scale, this distribution change appears almost horizontal. This status describes a uniform structure of the GCSN at medium-range and long-range distances, which may relate to a more linear than hierarchical structure and provide an aspect of the linear relationships empirically observed between global maritime trade and the economic size of its hinterland^7^. Taking into account that, in spatial networks, hubs undertake the majority of distance connectivity^3,6^, this value can be related to the existence of hubs. It can also loosely provide a characteristic spatial range of the hubs activity in the GCSN for distances over 3000 nm. On the other hand, the edge distribution of deadweight tonnage is short-tailed, with most of the connections having annual transport capacity up to 6 × 10^6^ tn. In the log–log scale, we can similarly observe a change in the edge distribution slope for annual transport capacity greater than 9 × 10^6^tn. In the metric scale, this distribution change also appears almost horizontal. In hub structure terms, this value of 9 × 10^6^tn expresses a characteristic value of the carrying capacity of the activity of hubs. Between these two cases of edge distribution, the slope of the distance-weighted edge distribution is smoother than this of tonnage-weighted. This difference implies that the hierarchical structure in the freight (DWT) distribution throughout the GCSN is more intense than its counterpart distance distribution. More intuitively, it describes that hubs proportionally undertake a higher freight transport load than serve distant transportation. This observation complies with the integration of the world economy and the lowering of border effects in maritime transport^18^.

### Hub connectivity as a function of distance

**Figure 3:**
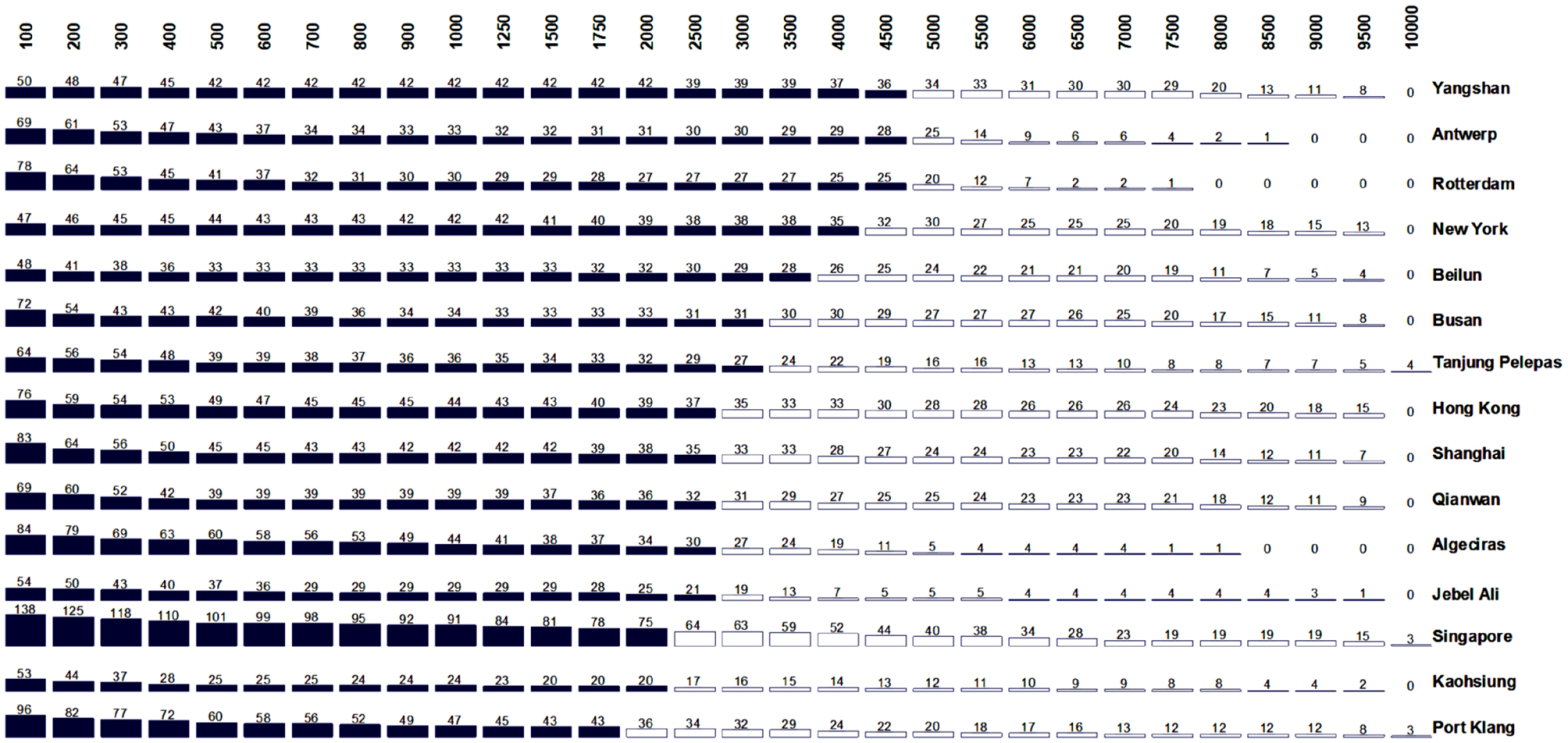
Bar charts of node degree across the available 32 GCSN layers (expressing node degree as a function of distance), for the selected ports shown in Table 2 (bar heights are proportional to the degree; dark bars represent values higher than row’s average).

This part of the analysis coordinates to the second dimension of the methodological framework, based on a decomposition rationale. It examines the connectivity performance of hubs, as a function of geographical distance, for the cases of highly connected ports shown in Table 2. The results are shown in Fig. 3, where it can be observed that the top ports can rank by their ability to deploy their connectivity over longer distances. Interestingly, ports with a dominance of gateway functions (import, export, sea-land transshipment, etc.) have longer-range connections, namely Yangshan, Antwerp, Rotterdam, and New York. The port of Yangshan is Shanghai’s new port, the largest of the country, serving the 26.3 million inhabitants global city and acting as a key point of entry for the Yangtze River port system^62^. New York is another global city on its own; and the second-largest container port in the U.S. after Los Angeles-Long Beach, with massive land transport connections reaching a vast hinterland^63^. Antwerp and Rotterdam have comparatively a much smaller city size but serve wide European hinterlands through multimodal connections. While Rotterdam has important transshipment functions as a hub for the British Isles, this activity only represents about 35% of its total activity. These four ports thus have in common a low level of transshipment incidence (below 50%), which is the share of containers shifted between vessels via the port terminals, with or without storage in between.

At the bottom of the figure are ports with a high level of transshipment incidence, exerting hub functions through hub-and-spokes and/or interlining configurations. This level reaches 80% for Singapore, meaning that only 20% of Singapore port’s activity is devoted to Singapore’s trade. A strong transshipment hub function implies a multiplication of high-frequency links between the hub and the feeder ports within a given region, thereby reinforcing short-range connectivity^64^. The geographic distribution of transshipment activity across the globe^64^ confirms that container hubs with a high transshipment incidence locate both along the round-the-world trunk line and at strategic passages such as the Gibraltar Straits (Algeciras), the Taiwan Straits (Kaohsiung) or are centrally located within a given region, such as Southeast Asia (Singapore) and the Persian Gulf (Jebel Ali).

These two groups echo the fundamental distinction between centrality and intermediacy proposed by the authors of^65^ about the spatial characteristics of transportation hubs. Ports like Rotterdam and New York have a high centrality, namely a strong traffic self-generation power derived from the accessibility to a vast market on the land side (hinterland). On the contrary, Singapore and Algeciras have a limited landward market and accessibility, relying mostly on intermediacy, namely the ability to embed the networks of transport operators for sea-sea transshipment. In fine, trade ports have longer-range connections due to the majority of direct deep-sea callings of containerships, while transshipment hubs, despite their global importance, have stronger connectivity locally as pivots through which many secondary ports consolidate their cargo to access the rest of the network.

Ports in between those two categories have a mixed profile, being at the same time hinterland ports and transshipment hubs, as seen with the cases of Busan and Hong Kong. Their interaction range is lower than gateway ports due to the importance of hub-and-spokes activities but higher than transshipment hubs since they also serve as gateways for a noticeable part of their activity. Busan, for instance, is South Korea's main port (80% of total container throughput) and second-largest city, serving the Seoul capital region distantly by land transport, while it also acts as a hub for numerous Japanese and Northern Chinese secondary ports^66^. Its transshipment share does not exceed 40%. Before its partial retrocession to China, Hong Kong had long been a transshipment hub like Singapore, with limited if no landside connectivity across the border^67^. Its transshipment activity had been a mix of Chinese re-exports and international transshipment (notably for Japan trade), combined with strong connections with Taiwan and, in particular, Kaohsiung until China-Taiwan direct trade had been relaxed across the Taiwan straits^68,69^. Afterward, Hong Kong gradually shifted its cargo handling operations to South China, evolving towards a global financial center with fewer transshipment activities^70^, and a growing gateway function serving the mainland Chinese hinterland.

This analysis shows that while larger ports tend to connect farther than smaller ports, trade (gateway) ports have a wider interaction range than transshipment ports. In some cases, this can be accentuated by geography, as seen with New York, which is relatively remote from the trunk shipping line connecting the Europe-Mediterranean area with the Caribbean basin. The latter region has become one important transshipment zone due to its proximity to the Panama Canal and its central position between North and South America. Most transatlantic flows are now rerouted through the Caribbean for consolidation. Similar observations could be made for other ports, such as in South Africa, which connections are longer on average due to peripherality^71^. Our results confirm a more generic process by which transshipment volumes (and shares) are higher as the deviation distance from the trunk line is lower^72^. Other factors also come into play such as urban size, hinterland connectivity, and port performance as a whole.

### Classification of links and ports

This approach builds on redistributing node strength (weighted degree) by percentiles of distance. One first result is striking, as the number and share of bidirectional links decrease with distance (Table 3), showing the importance of asymmetric pendulum services whereby ocean carriers deploy their vessels along with different loops between Europe-Asia and Asia-Europe^24^, for instance. Namely, the closer the ports the higher the probability for mutual interaction is, such as through short-sea, coastal, or feeder services. It is a corollary of the fact that trade decreases with distance, from an operational perspective. Such a result has no equivalent in network theory and its applications and can pave the way towards further research in all types of communication networks.

**Table 3:** Distribution of bidirectional links by distance percentiles.

Distance percentile	No. Links	Bi-directional links	% in total	% in percentile
1 (shortest)	1149	406	12.6	35.3
2	1150	393	12.2	34.2
3	1150	398	12.4	34.6
4	1149	354	11.0	30.8
5	1152	343	10.7	29.8
6	1149	328	10.2	28.5
7	1151	310	9.6	26.9
8	1150	279	8.7	24.3
9	1150	213	6.6	18.5
10 (longest)	1151	191	5.9	16.6
Total	11,501	3215	100.0	28.0

**Figure 4:**
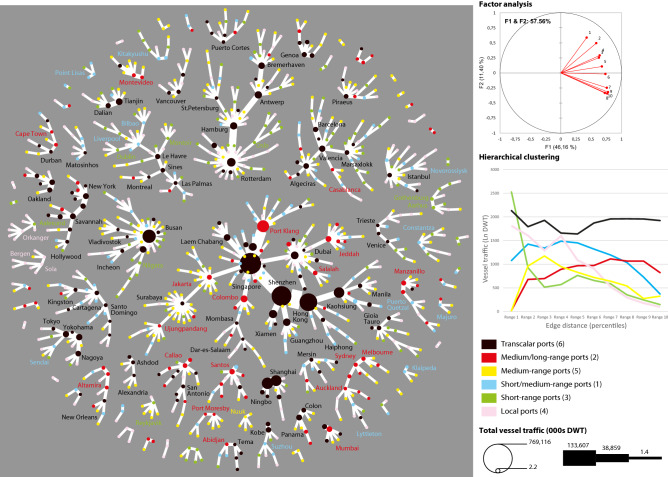
Multivariate analysis and visualization (space-L) of the GCSN (own elaboration based on TULIP 3.0.0; https://tulip.labri.fr/site/?q=node/110).

At next, factor analysis was launched based on ten variables, each of them representing the natural logarithm of traffic (DWT) by percentile of link distance (Fig. 4), from the shortest (distance percentile 1) to the longest (distance percentile 10). For all ports, their edges were classified into 10 classes based on their kilometric length. For each port, the variables thus correspond to total traffic per class. Namely, each variable represents the amount of traffic per port (in deadweight tons, DWT) along 10 classes of link distance. Those classes were defined based on the percentiles of distance, from the shortest to the longest links. Therefore, world ports will have more or less distant traffic based on this decomposition. Interestingly, factor 1 (horizontal) depicts a size effect by which all variables are projected on positive values. Factor 2 illustrates more a “scale effect” opposing shorter-range traffic (positive values) and longer-range traffic (negative values), with a gradual order from the shortest to the longest. A hierarchical clustering analysis considered the four main factors that concentrate 72% of the total variance with respective eigenvalue > 1. This analysis resulted in six clusters, each of them containing a relatively well-balanced number of ports. The nature of clusters was then revealed by visualizing their traffic distribution by percentile. Despite certain resemblances, each cluster has a specific distribution of traffic weight throughout the different distance ranges. Cluster#6 (including “trans-scalar” ports) handles the highest amount of traffic, Cluster#3 (short-range ports) manages traffic within ports’ vicinity, Cluster#4 (local ports) has the lowest traffic in the longest-range category, Cluster#2 (medium-, long-range ports), and #5 (medium-range ports) have nearly no local traffic, while Cluster#1 (short/medium-range ports) is an intermediary class.

A single linkage analysis algorithm was employed to bisect the graph, transform it into a tree, and reveal its main hubs, based on the hypothesis that hubs centralize all local, regional, and global level flows as distribution platforms^1^. Expectedly, Fig. 4 shows that hub-like structures mostly appear around black cluster ports (transcalar), among which are Singapore, Rotterdam, Valencia, Gioia Tauro, Istanbul, Piraeus, and New York, to name but a few. In addition, while such ports dominate their belonged subgraph, it is possible to observe a “range effect” whereby those subgraphs are organized as multi-hub structures, along a given maritime route. This is particularly the case of the largest component centered upon Singapore, which includes several other Asian hubs and gateways and even extends towards East Africa. Other trans-scalar Asian ports having a hub position appear as independent substructures, among which Busan (East Sea), Dalian-Tianjin (Yellow Sea), while main Japanese ports appear in relative isolation from the rest (Tokyo-Yokohama-Nagoya, Kobe-Osaka). The hub function is less developed for gateways as they are more specialized in sea-land transshipment ensuring direct trade, resulting in a lesser number of nodes in the star-like structures (e.g. Shanghai, Barcelona). The red cluster is the one with another noticeable number of hub-and-spokes configurations, differing from the black cluster by its limited local traffic. Ports in this category are often located in low-density port systems, such as Manzanillo (Central America West Coast), Callao (Peru), Australian main ports, Jeddah (Red Sea), Salalah (Arabian Peninsula), Makassar (Sulawesi), Casablanca, and Cape Town (South Africa), while their (relative) remoteness or their function as transshipment hub exacerbates their long-distance traffic. Santos and Montevideo are main national ports with strong transatlantic linkages, like Jakarta, Colombo, and Port Klang along the Europe-Asia route. Yet, Singapore's dominance remains overwhelming within this corridor. Other clusters are thus mostly composed of ports dominated by the previously mentioned hubs. For the medium-range cluster (yellow), the only exception is Nuuk in Greenland as a national hub. For local ports (light red), the three minor hubs are located in Norway, with a centrality also limited to the national (or even regional) scale. Short-range ports (green) as well are confined to North Europe, while short/medium-range ports (blue) are geographically diverse.

### Network measures as a function of distance

**Figure 5:**
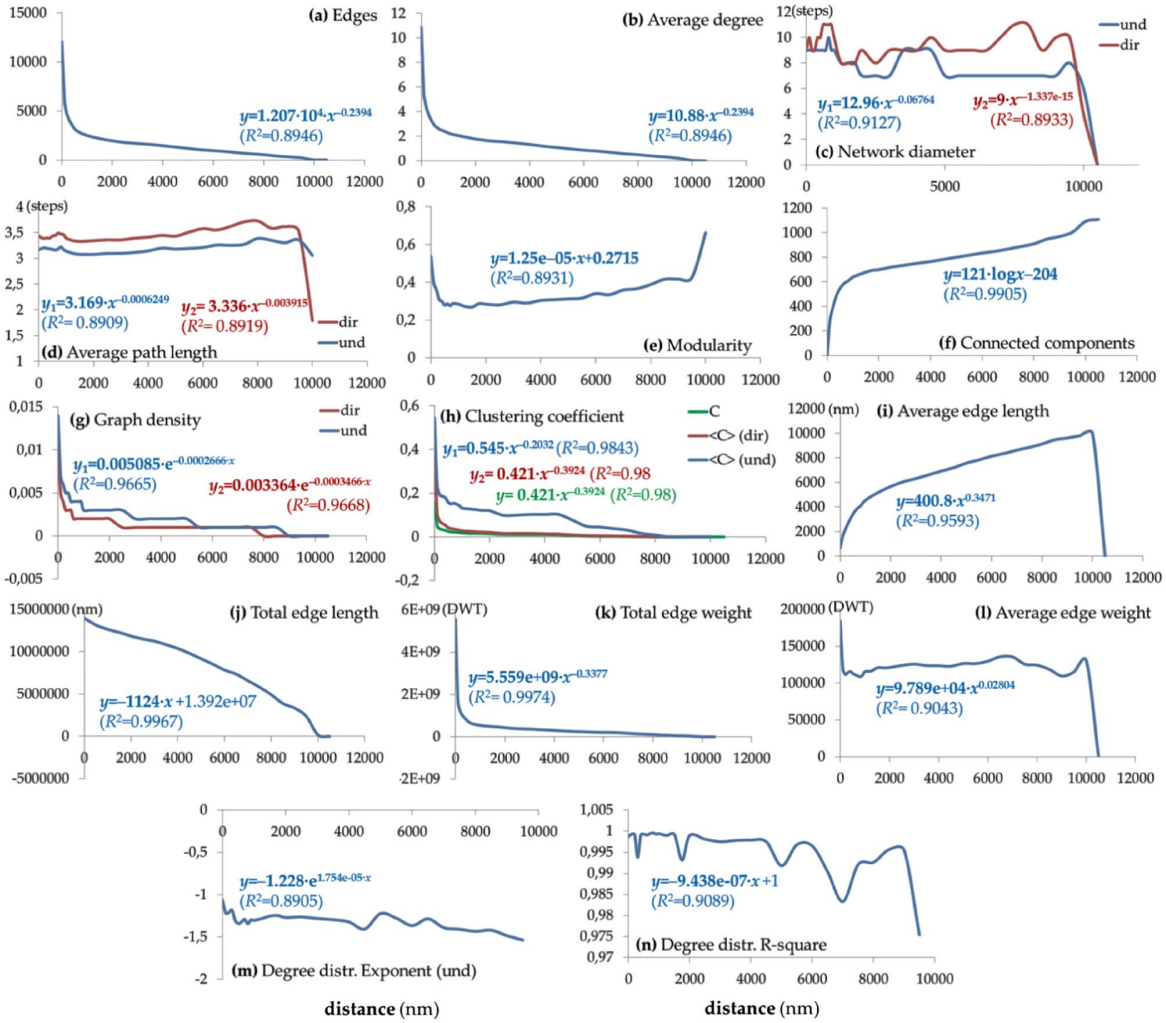
Line plots (metric scale) showing the distribution of major network measures of (**a**) network edges, (**b**) average degree (und), (**c**) network diameter (dir/und, measured in steps), (**d**) average path length (dir/und, measured in steps), (**e**) modularity, (**f**) number of connected components, (**g**) graph density (dir/und), (**h**) clustering and average clustering coefficient (dir/und), (**i**) average edge length (nm), (**j**) total edge length (nm), (**k**) total edge weight (DWT), (l) average edge weight (DWT), (**m**) PL degree distribution exponent (und), and (**n**) PL degree distribution determination (R^2^), which are expressed as a function of distance and computed on the series of layers composing the multilayer model of GCSN (as shown in Fig. 1). Equations of the best possible (those with max *R*^2^) fitting curves are shown per diagram.

This part of the analysis builds on the decomposition rationale shown in relation 2, where the GCSN topology is composed of several topological features expressed as a function of distance. The results are shown in Fig. 5, where we can observe that the measures of (number of) edges (Fig. 5a), average degree (Fig. 5b), clustering and average clustering (Fig. 5h) coefficient, and total edge weight (Fig. 5k) are all described by a decaying (PL-like) pattern. This PL configuration expresses a decline of these measures as distance increases. In terms of clustering, this pattern illustrates that circular (triangular) connections become less in number as distance decreases and thus that more linear structures in the connectivity of GCSN emerge. Next, the patterns of network diameter (Fig. 5c) and average path length (Fig. 5d) appear “noisy constant”, described by a horizontal slope equipped with several local fluctuations that generally imply a relative indifference of the path-defined GCSN measures to distance. However, these local fluctuations may reveal levels of geographical scale where the path-determined network topology is either consistent or asymmetric. For instance, at the range of 6500–8500, we can observe a concave area in the directed aspects of both measures, implying more intense asymmetric forces in the topology of GCSN.

On the other hand, the measures of average edge length (Fig. 5i) and average edge weight (Fig. 5l) are described by ascending PL patterns, which impressively illustrate that the average trade volume carried throughout the GCSN appears indifferent to distance, for distances greater than 100 nm. The number of connected components (Fig. 5f) also follows an ascending pattern, implying that the connectedness of the GCSN decomposes as distance increases, but this effect is more intense at smaller distances (< 1000 nm) and becomes smoother at greater ones. Next, the patterns of graph density (Fig. 5g), degree distribution exponent (Fig. 5m), and coefficient of determination *R*^2^ (Fig. 5n) are decaying exponential ones, illustrating a decline through distance. For the degree distribution exponent, this decaying pattern illustrates that the topology of the GCSN has more distinctive hub-and-spoke characteristics as distance increases. Next, modularity (Fig. 5e) configures a “U”-shaped pattern, illustrating a general increase of the measure through distance, but not at the neighborhood (< 300 nm) and large (< 8500 nm) scale. This shape implies a tendency of the GCSN to separate into communities due to local and distant connectivity. Finally, the total edge length (Fig. 5j) configures a linear declining pattern, implying that large distances play an important role in the cohesion of the network, as imprinted on the uniform decay of the total edge length.

**Figure 6:**
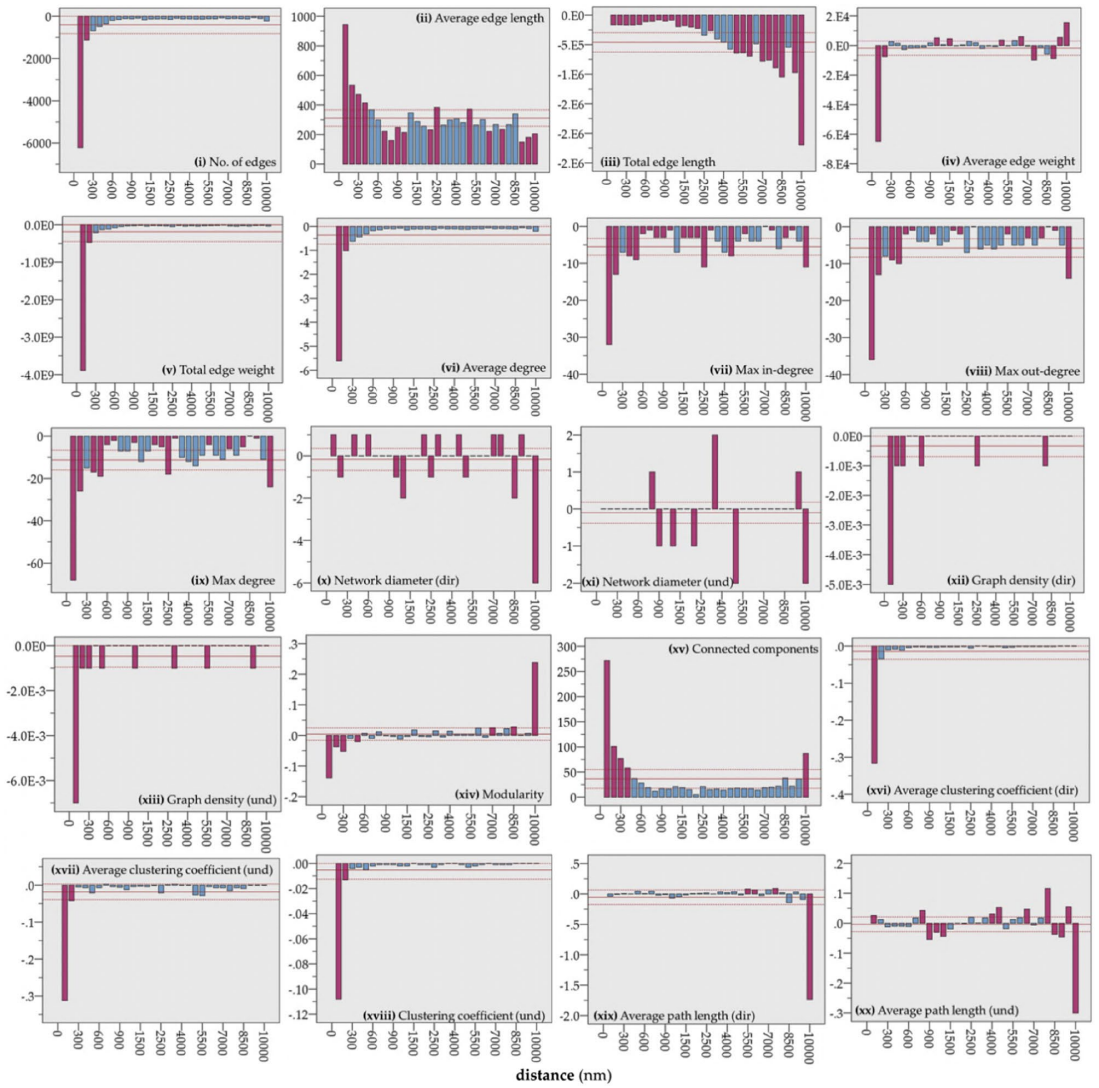
Bar plots (categorical axis) showing the first-order differences of the GCSN’s attribute-series of (**i**) network edges, (**ii**) average edge length (nm), (**iii**) total edge length (nm), (**iv**) average edge weight (DWT), (**v**) total edge weight (DWT), (**vi**) average degree, (**vii**) max in-degree, (**viii**) max out-degree, (**ix**) max degree, (**x**) network diameter (und, steps), (**xi**) network diameter (dir, steps), (**xii**) graph density (dir), (**xiii**) graph density (und), (**xiv**) modularity, (**xv**) connected components, (**xvi**) average clustering coefficient (dir), (**xvii**) average clustering coefficient (und), (**xviii**) clustering coefficient (und), (**xix**) average path length (und, steps), (**xix**) average path length (und, steps), which are expressed as a function of distance and are computed on the series of layers composing the multilayer model of GCSN (as shown in Fig. 1).

To examine topological changes between successive layers of GCSN and thus across different levels of geographical distance, we apply a first-order differences analysis, according to the relation 1. The results of the analysis are shown in Fig. 6, which illustrates significant changes in measures of network topology across the available distance levels. As it can be observed, a significant amount of the GCSN edges (Fig. 6i) is distributed at the neighborhood scale (< 200 nm). For average edge length (Fig. 6ii), up to a range of 400 nm, and at the distances of 2500 nm and 5500 nm, changes are significantly greater than the mean. However, the total edge length (Fig. 6iii) shows significantly great changes at distances greater than 5000 nm. For average edge weight (Fig. 6iv), we can observe three zones, a neighborhood (0–200 nm), a middle (“mesoscale”) geographical scale (1000–1500 nm), which perhaps describes international market regions, and a large (mega) geographical scale (≥ 5000 nm). This allows defining three different types of markets in the GCSN, according to the average freight distribution and to the geographical scale: the *neighborhood*, the *international*, and the *intercontinental* market. For the total edge weight (Fig. 6v), significant changes appear at the level of the neighborhood, highlighting the importance of the neighborhood market in the GCSN. For average degree (Fig. 6vi), a decaying pattern is evident implying that a significant number of routes in the GCSN are distributed within the scale of the neighborhood. Next, for the max in-degree (Fig. 6vii), we can observe significantly high changes at distances < 500 nm and locally at 2500 nm, 4500 nm (mesoscale), and 10,000 nm (large or mega-scale). This distancing can apply another zoning in terms of importing maritime market: the neighborhood (< 500 nm), the international (2500–4500 nm), and the intercontinental market (10,000 nm).

For the max out-degree (Fig. 6viii), the neighborhood (< 500 nm) and intercontinental (10,000 nm) zones are also evident, but not the middle (mesoscale) zone. The difference in the patterns between max in- and out-degree implies that export trade occurs at larger distances than import trade. This observation can be verified by applying an independent samples *t* test for the equality of means between the upper (out-degree) and lower triangular (in-degree) distance weights matrix of the GCSN. This observation provides evidence about the asymmetric structure of the GCSN functionality at the mesoscale zone. Next, network diameter shapes a rather variable picture, both for its directed (Fig. 6x) and undirected (Fig. 6xi) expressions. Its pattern implies that this measure is more dependent on mesoscale and large-scale connections. A variable decaying pattern also describes graph density (Fig. 6xii,xiii). Next, the pattern of modularity (Fig. 6xiv) also supports that significant changes occur at the neighborhood and large scales. The patterns of clustering (Fig. 6xviii) and average clustering coefficient (Fig. 6xvi,xvii) illustrate that clustering in the GCSN is mainly a matter of neighborhood connections and thus is related to the dynamics of the local markets. Finally, the average path length (Fig. 6xix,xx) produces zoning at the neighborhood scale (100 nm), the very beginning (800–1250 nm), and the end of the middle scale (4500–5500 nm), and the large scale (≥ 6500 nm).

**Figure 7:**
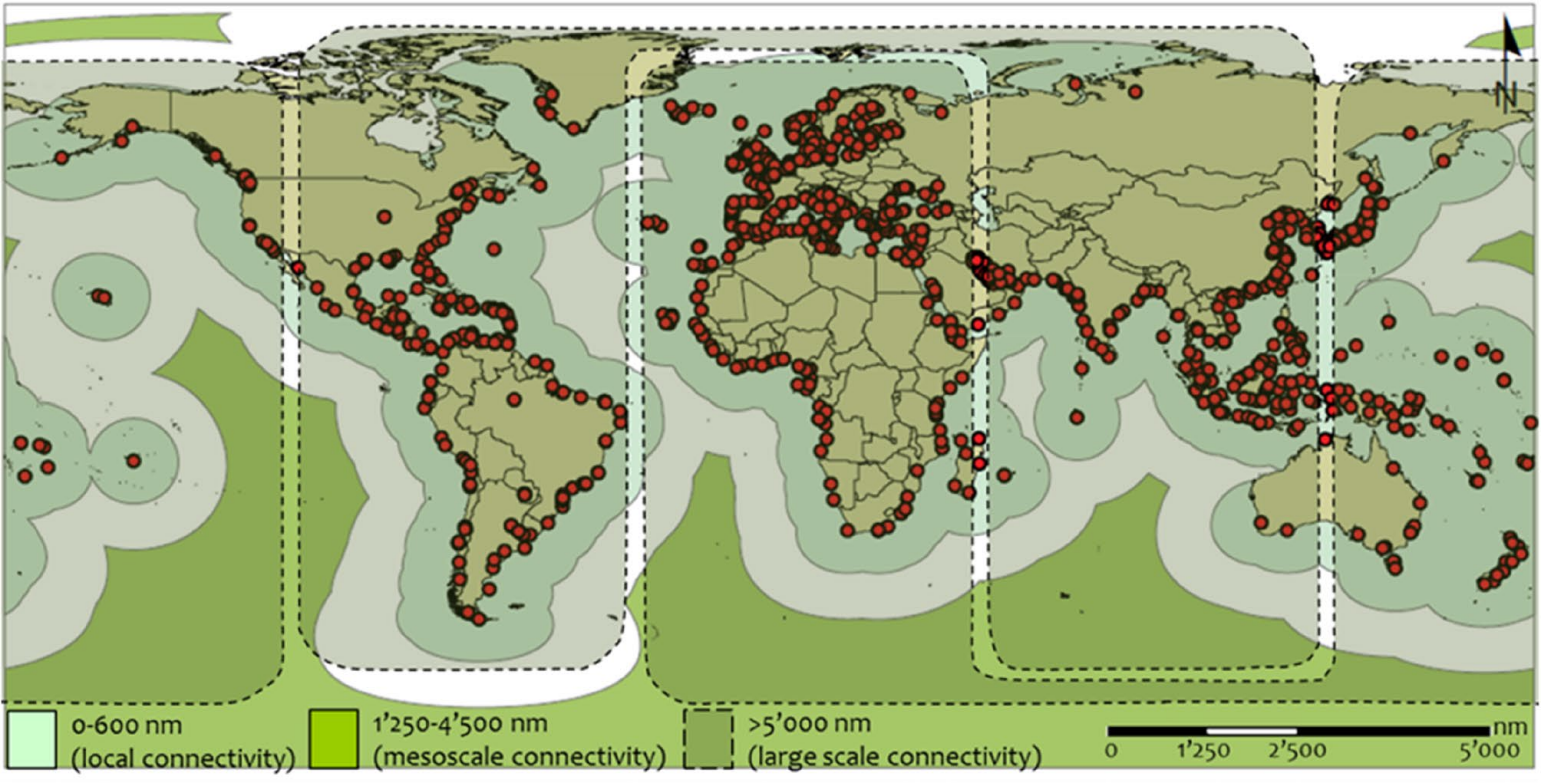
Zone buffering of the geographical scales (local, mesoscale, and large connectivity) of the GCSN functionality, as revealed from the analysis (own elaboration based on ESRI ArcGIS 10.50; https://www.arcgis.com).

An attempt to combine all the previous observations to a single one results in three levels of geographical scale in the structure and functionality of GCSN. These scales are (a) the neighborhood (local) connectivity scale, defined by distances shorter than 600 nautical miles (< 600 nm), (b) the international (mesoscale or middle) connectivity scale, defined by distances at the range of 1250–4500 nm, and (c) the market of intercontinental (large) connectivity scale, defined by distances greater than 5000 nautical miles (> 5000 nm). These geographical scales are visualized into buffers at the map of Fig. 7.

## Conclusions

This paper examined how spatial distance affects the Global Container Shipping Network (GCSN) topology, using a multilayer network model consisting of 32 multiplex layers generated by successively removing shorter distance connections. The degree and edge distribution analysis showed that the structures of hierarchy in the GCSN are more intense and distinguishable per trade role (import and export). When the GCSN is interpreted under the trade balance, these directed structures of hierarchy appeared more counterbalanced. Also, it showed that the hierarchical structure in the freight distribution of the GCSN is more intense and homogenized than the distance distribution. The hubs connectivity showed a significant loss at shorter distances (≤ 800 nm) and a smoother decay at longer distances. Trade/gateway hubs like Antwerp, Rotterdam, and Yangshan have a higher interaction range than transshipment hubs such as Singapore, Algeciras, and Kaohsiung, which connectivity is more local due to the high frequency and multiplication of local linkages with feeder ports. A balanced profile was detected for Busan and Hong Kong, which are both gateway (hinterland) ports and hub (transshipment) ports. Also, the analysis revealed that bidirectional links in GCSN decrease with distance, highlighting the importance of asymmetric functionality in the carriers’ operations. According to their distribution of traffic over classes of distance, ports were classified into six clusters (local, short-range, short/medium-range, medium-range, medium/long-range, and trans-scalar ports). Transcalar ports dominate the network when the latter is transformed into a tree-like structure revealing hubs and their belonged subgraphs. At last, the analysis on network measures verified the scale-free-like topology of the GCSN and illustrated a linear configuration of the GCSN in higher distances. The path-defined measures and the average trade load showed a relative indifference to distance, supporting the empirical finding of the integration of the world economy and maritime transportation. The connectedness of the GCSN was also found to decompose as distance increases, but mainly at smaller distances (< 1000 nm). Finally, the analysis revealed in the structure of GCSN 3 levels of geographical scale, configuring the (a) neighborhood (local connectivity, < 600 nm), (b) international (mesoscale or middle connectivity, 1250–4500 nm), and (c) intercontinental (large scale connectivity, > 5000 nm) markets.

The overall approach contributed to the study of the spatial dimension in complex and multilayer networks and provided insights into the spatial structure of the GCSN maritime market. Especially the identification of transcalar nodes is an addition to the literature on hub theory^73^, as it demonstrates that such nodes dominate through their ability to connect all traffic scales, compared with recent studies of the GCSN only focusing on topology^74^. The directional decay over distance is another new finding, which can serve as a benchmark to study any other domain subject to asymmetrical relationships, from trade imbalance to empty container repositioning and power structures in social and firms networks.
